# Evolution of Antimicrobial Resistance in *Klebsiella pneumoniae* over 3 Years (2019–2021) in a Tertiary Hospital in Bucharest, Romania

**DOI:** 10.3390/antibiotics13050431

**Published:** 2024-05-10

**Authors:** Alexandra Cireșă, Daniela Tălăpan, Carmen-Cristina Vasile, Cristina Popescu, Gabriel-Adrian Popescu

**Affiliations:** 1 Faculty of Medicine, “Carol Davila” University of Medicine and Pharmacy, 050474 Bucharest, Romaniacarmen-cristina.vasile@drd.umfcd.ro (C.-C.V.); cristina.popescu@umfcd.ro (C.P.);; 2 “Prof. Dr. Matei Balș” National Institute of Infectious Diseases, 021105 Bucharest, Romania

**Keywords:** multidrug-resistant *Klebsiella pneumoniae*, carbapenem-resistant *Klebsiella pneumoniae*, risk factors for antimicrobial resistance

## Abstract

Background: The antimicrobial resistance (AMR) of *Klebsiella pneumoniae* recorded a steep upward trend over the last two decades, among which carbapenem-resistant *Klebsiella pneumoniae* (CRKP) is one of the most concerning strains considering the development and spread of AMR. The aim of this study was to analyze the evolution of AMR for *Klebsiella pneumoniae* and to describe the risk factors of AMR for *Klebsiella pneumoniae*, including the COVID-19 pandemic. Methods: We conducted a retrospective study on *Klebsiella pneumoniae* non-duplicative isolates collected from patients admitted to a tertiary hospital in Bucharest, Romania, from January 2019 to December 2021. We evaluated AMR changes by comparing resistance between 2019 and the mean of 2020–2021. Results: The rates of AMR increased for third-generation cephalosporins, carbapenems, aminoglycosides, fluoroquinolones, and colistin and decreased for trimethoprim/sulfamethoxazole (TMP/SMX), 45.7% in 2019 vs. 28.3% in 2021. A longer length of hospital stay (ꭓ^2^ = 49.68, *p* < 0.01); recent antibiotic consumption, RR = 1.38, 95% CI [1.21, 1.57]; and recent contact with hospital settings, RR = 1.54, 95% CI [1.32, 1.8] were risk factors for multidrug-resistant (MDR) *Klebsiella pneumoniae*. Conclusions: The AMR of *Klebsiella pneumoniae* increased during 2020–2021 for most of the potential active antibiotics; only TMP/SMX resistance decreased, and it may represent a treatment option for CRKP or MDR *Klebsiella pneumoniae* infections. Decreasing the excessive use of antibiotics and the implementation of prevention and control measures in healthcare settings are mandatory for avoiding further increases in the AMR rate of *Klebsiella pneumoniae*.

## 1. Introduction

*Klebsiella pneumoniae* is a Gram-negative bacillus that can colonize the human body, especially the gastrointestinal tract, but may also be involved in a wide range of human infections. *Klebsiella pneumoniae* produces community-acquired infections and healthcare-associated infections (HAIs) such as urinary tract infections, pneumonia, intra-abdominal infections, blood stream infections, and skin and soft tissue infections [1,2,3].

Over the last 20 years, the antimicrobial resistance (AMR) of *Klebsiella pneumoniae* has increased rapidly worldwide, as evidenced by high rates of multidrug-resistant (MDR) *Klebsiella pneumoniae* [4]. For this reason, *Klebsiella pneumoniae* was included in the ESKAPE group of MDR pathogens (*Enterococcus faecium*, *Staphylococcus aureus*, *Klebsiella pneumoniae*, *Acinetobacter baumannii*, *Pseudomonas aeruginosa*, and *Enterobacter* spp.), which are frequently involved in HAIs [5]. Regarding antibiotic-resistant bacteria, in 2017, the World Health Organization (WHO) published a list of priority pathogens—divided into three groups: critical, high, and medium priority—for research and development of new antimicrobials. Carbapenem-resistant, ESBL-producing *Klebsiella pneumoniae* has been placed among the critical pathogens, along with other carbapenem-resistant, ESBL-producing Enterobacterales (such as *Escherichia coli*, *Serratia* spp. and *Proteus* spp.), carbapenem-resistant *Acinetobacter baumannii,* and carbapenem-resistant *Pseudomonas aeruginosa* [6].

*Klebsiella pneumoniae* has developed resistance on various levels against all categories of potentially active antibiotics, including third-generation cephalosporins (C3G), carbapenems, aminoglycosides (AG), fluoroquinolones (FQ), colistin, trimethoprim/sulfamethoxazole (TMP/SMX), and the newer antibiotics carbapenems/beta-lactamase inhibitors and cefiderocol [4,7,8,9].

Carbapenem-resistant *Klebsiella pneumoniae* (CRKP) represents one of the most concerning problems within the development and spread of AMR worldwide. In order to assess the spread of AMR for bacterial species that may have clinical and epidemiological impact, in 2011, the European Center for Disease Prevention and Control (ECDC) founded the European Antimicrobial Resistance Surveillance Network (EARS-Net), which collects and analyzes data about invasive infections produced by *Klebsiella pneumoniae*, *Acinetobacter* spp., *Escherichia coli*, *Enterococcus faecalis*, *Enterococcus faecium*, *Staphylococcus aureus*, *Streptococcus pneumoniae* and *Pseudomonas aeruginosa* from countries in the EU/EEA. According to EARS-Net, the population-weighted mean of carbapenem resistance for *Klebsiella pneumoniae* increased from 7.9% to 11.7% from 2019 to 2021 in the EU/EEA. Among the EU/EEA countries, the greatest rates of CRKP are reported in Greece, Romania, Italy, and Bulgaria. The level of carbapenem resistance for *Klebsiella pneumoniae* in Romania increased from 32.3% in 2019 to 54.5% in 2021 [10].

The carbapenem resistance of *Klebsiella pneumoniae* is mainly mediated by the production of several types of carbapenemases [11], among which a major treatment problem is represented by metallo-beta-lactamases (MBL) [12]. In recent years, for isolates of *Klebsiella pneumoniae* that produce other types of carbapenemases (i.e., KPC, OXA-48), new cephalosporin/carbapenem-beta lactam inhibitor antibiotics have been approved; still, for MBL-producing *Klebsiella pneumoniae* infections, treatment options amongst beta lactam antibiotics are limited to aztreonam and cefiderocol [11,13]. Available antimicrobial treatment options in a MBL-producing *Klebsiella pneumoniae* infection consist frequently of colistin, fosfomycin or tigecycline, along with the two beta lactam antibiotics mentioned above [14,15]. Some data show that CRKP infections have higher rates of mortality than carbapenem-susceptible *Klebsiella pneumoniae* (CSKP) infections [2,16].

Risk factors for CRKP infections include prior antibiotic exposure (especially carbapenems), immunosuppression, intensive care unit admission, mechanical ventilation, central venous catheterization, surgery, and nasogastric intubation [16].

The current study aims to analyze the AMR changes of *Klebsiella pneumoniae* and to describe risk factors for *Klebsiella pneumoniae* AMR resistance using isolates collected between January 2019 and December 2021 from a tertiary hospital in Bucharest, Romania.

## 2. Results

### 2.1. Demographic Features

The isolates of *Klebsiella pneumoniae* included in the study were collected from a total number of 706 patients—381 patients in 2019, 151 patients in 2020 and 174 patients in 2021. The isolates of *Klebsiella pneumoniae* were collected from 376 (53.3%) women and 330 (46.7%) men. The mean age was 56.9 years (SD = 21.44) for 2019, 56.4 years (SD = 20.63) for 2020, and 59.8 years (SD = 17.04) for 2021. Age showed asymmetrical distribution for all 3 years (Kolmogorov–Smirnov test; D(381) = 0.12, *p* < 0.01 for 2019; D(151) = 0.11, *p* < 0.01 for 2020; D(174) = 0.1, *p* < 0.01 for 2021), and there was no significant difference found when comparing mean age between years [one-way ANOVA, F(2,703) = 1.46, *p* = 0.23].

### 2.2. Epidemiological and Clinical Features

The bacterial isolates included in the study were collected from 161 outpatients and 545 inpatients. For the inpatients, the samples of *Klebsiella pneumoniae* were isolated in the first 3 days of hospitalization for 306 (56.1%) patients, between 3 and 10 days of hospitalization for 99 (18.2%) patients and after more than 10 days of hospitalization for 140 (25.7%) patients. A total of 366 (51.8%) isolates of *Klebsiella pneumoniae* had an MDR profile; 671 isolates of *Klebsiella pneumoniae* were tested for carbapenem resistance, of which 232 were CRKP. All CRKP isolates for which we performed antibiotic susceptibility testing had an MDR profile. The frequency of MDR and CRKP *Klebsiella pneumoniae* were higher in inpatients, as shown in Table 1. The AMR profiles’ distribution for the isolates included in the study is mentioned in Table 1.

The frequencies of CRKP and MDR *Klebsiella pneumoniae* were higher for samples collected later during hospitalization compared to those collected in the first days of hospitalization, as shown in Table 2.

The isolates of *Klebsiella pneumoniae* included in the study were collected from patients aged between 0 and 94 years-old, mostly from the 61–80 years group. Table 3 shows the associations between these age groups and different AMR profiles of *Klebsiella pneumoniae*.

Recent contact with hospital settings and recent antibiotic usage are both risk factors for MDR *Klebsiella pneumoniae* infection/colonization. Recent antibiotic usage is also a risk factor for CRKP infection/colonization, as shown in Table 4.

### 2.3. Antimicrobial Resistance of Klebsiella pneumoniae

The rate of resistant *Klebsiella pneumoniae* isolates was significantly higher in 2020–2021 compared to 2019 for all tested antibiotics except TMP/SMX and fosfomycin (Table 5). Table 5 presents the evolution of AMR for *Klebsiella pneumoniae* between 2019 and 2021 for different classes of antibiotics and for MDR *Klebsiella pneumoniae*. Table 5 also includes the evolution of MBL-producing *Klebsiella pneumoniae* between 2019 and 2021, calculated as a percentage from the CRKP isolates.

Regarding resistance to aminoglycosides, 142 (56.1%) isolates of *Klebsiella pneumoniae* defined as resistant to aminoglycosides showed preserved susceptibility to one aminoglycoside. Of those 142 isolates of *Klebsiella pneumoniae*, 134 were resistant to gentamycin and susceptible to amikacin, and 8 were susceptible to gentamycin and resistant to amikacin.

Data regarding the antimicrobial resistance of CRKP to colistin, TMP/SMX, fosfomycin, and tigecycline are presented in Table 6.

## 3. Discussion

*Klebsiella pneumoniae* is one of the main pathogens with extensive AMR profiles that include carbapenem resistance. CRKP infections are likely to have severe clinical outcomes [16]. Additionally, some data show that the mortality induced by CRKP infections seems to be higher than the mortality associated with CSKP [2,17,18,19]. In order to provide a hypothetical prevention and control plan for CRKP infections, Li J. et al. conducted a meta-analysis for the purpose of exploring risk factors for CRKP infections. Their results show that prior antibiotic exposure increases the risk of CRKP infections, while age is not associated with CRKP infections [16]. According to a study conducted by Jiao Y et al., even though age was not found to be a risk factor for CRKP infection/colonization, age seems to be an independent risk factor of mortality in patients with CRKP infection/colonization [20]. In our study, age between 41 and 80 years old was found to be a risk factor for CRKP infection. Another meta-analysis published by Liu P. et al. showed that alongside previous antibiotic use, prior hospitalization and a longer length of hospital stay are risk factors for CRKP infections [21]. Regarding MDR *Klebsiella pneumoniae*, a cross-sectional study lead by Ibrahim ME pointed out that patients aged 65 years or older are at higher risk of developing MDR *Klebsiella pneumoniae* infections compared to younger patients [22]. Our results show that age ≥ 61 years old is a risk factor for MDR *Klebsiella pneumoniae* infections. According to our data, recent antibiotic usage is a risk factor for CRKP and MDR *Klebsiella pneumoniae* infections, and recent contact with hospital settings is a risk factor for MDR *Klebsiella pneumoniae* infections. However, the impact of recent antibiotic usage and recent contact with hospital settings might be underestimated due to lack of data from some of the patients’ medical histories. Moreover, recent contact with hospital settings seems unlikely not to be associated to CRKP infections. We consider that the lack of association might be determined by the small number of cases for which we had information about prior hospitalizations.

We found higher rates of CRKP and MDR *Klebsiella pneumoniae* among inpatients compared to outpatients. A study conducted in China by Ho Y et al., who analyzed data about risk factors for CRKP, found higher odds of isolating imipenem-resistant *Klebsiella pneumoniae* in samples collected from inpatients than from outpatients [23]. In addition, we observed an increase in frequencies of CRKP and MDR *Klebsiella pneumoniae* with longer hospitalization. Therefore, longer exposure to hospital settings increases the risk of colonization or HAIs produced by CRKP or MDR *Klebsiella pneumoniae*.

Regarding the AMR of *Klebsiella pneumoniae*, EARS-Net is collecting data about resistance to third-generation cephalosporins, carbapenems, aminoglycosides, fluoroquinolones, and the combined resistance to some of these antibiotics. This surveillance system receives data from all the EU member states (including Romania) and some of the EEA countries [10].

For the 2020–2021 period, we found higher levels of resistance for *Klebsiella pneumoniae* to C3G, carbapenems, AG, FQ and colistin compared to 2019. These differences are probably because in 2020–2021, most of the infections produced by *Klebsiella pneumoniae* (as well as by other bacteria) were hospital-acquired, considering that the main diagnosis upon admission was COVID-19. Instead, in 2019, *Klebsiella pneumoniae* infections were more often community-acquired, produced by wild-type strains of *Klebsiella pneumoniae*, with lower AMR profiles.

According to EARS-Net, the population-weighted mean of resistance to C3G for *Klebsiella pneumoniae* had an approximatively constant level of about 34% from 2019 to 2021 in the EU/EEA [24]. In Romania, on the other hand, the levels of resistance to C3G for *Klebsiella pneumoniae* were about two-fold higher during the same period of time, reaching 70.8% in 2021 [10]. The level of resistance reported in our study is lower compared to the Romanian national data by 13.8% in 2019, 8.8% in 2020 and 9.1% in 2021. Other than hospital-to-hospital AMR differences, our study included all isolates of *Klebsiella pneumoniae* collected in the hospital in the given period of time, not only invasive isolates, as it is in the national surveillance system [25].

In terms of carbapenem resistance, the level of resistance reported to EARS-Net from Romania were 32.3% in 2019, 48.3% in 2020, and 54.5% in 2021 [10,26]. Romania exceeded the European weighted mean of carbapenem resistance by over four-fold in 2020–2021, meaning it was in second place (after Greece) among the European countries with high levels of resistance in 2019–2021 [10]. Our findings are lower than the levels reported by Romania in 2019 and 2020 and higher than in 2021. We believe that these differences occurred because in 2019 and 2020, some of the patients had community-acquired *Klebsiella pneumoniae* infections, while in 2021, when the hospital was dedicated to patients with COVID-19, the number of HAIs produced by *Klebsiella pneumoniae* was higher as they occurred during hospitalization. The carbapenem resistance of *Klebsiella pneumoniae* is mainly determined by production of carbapenemases [11], of which metallo-beta-lactamases have a scarcity of treatment options [14,15]. Our study found a more than 10-fold increase in MBL-producing isolates between 2019 and 2020–2021. Additionally, carbapenem-producing Gram-negative bacteria (including *Klebsiella pneumoniae*) have been found in the rectal carriages of asymptomatic patients in Romania, as reported by Timofte et al. in 2016 [27]. Outbreaks of MBL-producing *Klebsiella pneumoniae* have been reported in several countries in Europe between 2019 and 2021, and the most frequently reported MBL was NDM (New Delhi metallo-beta-lactamase) [28,29,30]. The national registers of France presented an increasing proportion of NDM-producing Enterobacterales from 8.6% to 23.9% of the total number of carbapenem-resistant Enterobacterales between 2014 and 2021 [31]. Furthermore, in recent years, attention has been drawn to the emergence and spread of strains of *Klebsiella pneumoniae,* producing associations of carbapenemases [11]. The first isolation of Enterobacterales co-harboring NDM and OXA-48 genes in Romania was reported in 2013 [32].These associations represent a serious red flag regarding future perspectives on antimicrobial resistance, considering that treatment options for these types of bacteria are even more limited.

The rates of aminoglycoside-resistant *Klebsiella pneumoniae* in our hospital were lower than the national rates in 2019, being 29% in our study vs. 53% in the Romanian national reports, but we found comparable rates of resistance in 2020 and 2021 [10]. These data show the level of resistance to at least one aminoglycoside. However, about half of the isolates that were reported resistant to aminoglycosides in our hospital had preserved susceptibility to one aminoglycoside, with most of them being susceptible to amikacin. Therefore, aminoglycosides may remain a viable treatment option for *Klebsiella pneumoniae* infections, especially for upper urinary tract infections. This treatment option is of particular interest because gentamycin and amikacin show lower resistance and lower incidence of *Clostridioides difficile* infection than antibiotics from other groups [33]. The rates of fluoroquinolone-resistant *Klebsiella pneumoniae* collected in our study ranged from 49.7% to 64.9%, which was below the national average of 62% to 67.2% for 2019–2021. For both aminoglycosides and fluoroquinolones, our findings and national levels were much higher than the EU/EEA-reported resistance in the same period of time, ranging from 23.7% to 24.5% for aminoglycosides and from 33.6% to 34% for fluoroquinolones [10,24].

The MDR profile of *Klebsiella pneumoniae*, recorded in European and national surveillance systems as combined resistance to C3G, AG and FQ, had an approximately constant level of about 21% in the EU/EEA in 2019–2021. In Romania, a slight decrease from 52% to 48.4% between 2019 and 2021 was registered [10,24]. Our findings are similar to national levels for 2019, but we recorded an increase of about 10% from 2019 to 2021.

Alongside data related to antibiotics reported by EARS-Net, we collected data about four other antibiotics, TMP/SMX, fosfomycin, colistin and tigecycline, for which we also described the resistance trends among CRKP isolates. We found that the rate of TMP/SMX resistance for CRKP isolates was about two-fold lower in 2021 compared to 2019. A similar study conducted in China from 2014 to 2022 found an approximately stable level of resistance to TMP/SMX of about 23% for *Klebsiella pneumoniae* isolates but reported an increase in the resistance rate to TMP/SMX for CRKP isolates between 2019–2021, from 10.5% to 65.6% [34]. Another study conducted in China showed a relatively constant rate of TMP/SMX resistance for *Klebsiella pneumoniae* isolates of about 24% from 2017 to 2020 [35].

The Romanian laboratories reporting to EARS-Net also provide data about colistin resistance [25]. We believe that testing antimicrobial susceptibility to colistin for CSKP isolates should not be advised because there might be a risk of encouraging the excessive use of colistin. The same attitude is also justified for intravenous fosfomycin and tigecycline. We will further refer only to colistin resistance in CRKP isolates. In Romania, the rates of resistance to colistin increased from 29.1% to 36.7% between 2019 and 2021 for CRKP isolates [25,36]. An ascending trend of colistin resistance from 14.9% in 2016 to 36.2% in 2021 was also described for CRKP isolates by Shein AMS et al. in a study conducted in a hospital in Thailand [37]. Our data indicated an increasing rate of colistin resistance, from 54.7% to 80% for CRKP isolates, between 2019 and 2021.

The number of *Klebsiella pneumoniae* isolates for which intravenous fosfomycin susceptibility testing was performed in our laboratory was radically reduced between 2019 and 2021, and the isolates tested in 2021 were mostly CRKP isolates. The rate of fosfomycin resistance for CRKP isolates increased between 2019 and 2021, as in 2021 almost all the tested CRKP isolates were resistant to fosfomycin. However, because the number of CRKP isolates which were tested for susceptibility to fosfomycin and tigecycline was relatively low, we do not rely on the applicability of the statistical analysis, and only descriptive data were presented. A study of AMR trends for *Klebsiella pneumoniae* which was conducted in a hospital in Italy found that there was an average of 24.6% resistance to fosfomycin from 2016 to 2020 for all isolates of *Klebsiella pneumoniae* [38].

In 2019, our hospital did not report any CRKP isolate with an MIC > 0.5 mg/L for tigecycline, while in 2020 and 2021, we had seven such CRKP isolates. While testing susceptibility to tigecycline might seem irrelevant for *Klebsiella pneumoniae* at a time when there is no established breakpoint of resistance in the EUCAST recommendations [39], we continue to perform it in cases wherein the AMR of *Klebsiella pneumoniae* reaches profiles such as extensively drug-resistant (XDR) and pan-drug-resistant (PDR), because in many cases, we might not have available treatment options for infections produced by these strains of *Klebsiella pneumoniae*. A study conducted in China which was published in 2022 found that there was a reduction in the resistance rates of tigecycline for *Klebsiella pneumoniae* between 2017 and 2020, from 2.4% to 0.6%. This Chinese study used the Clinical and Laboratory Standards Institute (CLSI) recommendations for MIC interpretation to tigecycline [35].

### Study Limitations

One of the limitations is represented by the differences in number of isolates between 2019 and 2020–2021. The main reason for this is the smaller number of patients admitted during the COVID-19 pandemic. We had a small amount of data in the analysis of risk factors (recent antibiotic use and recent contact with hospital settings) because of a lack of data in the patients’ medical histories, which were taken from an electronic database. Another limitation of our study could be the single-center data collection. However, our hospital was the main facility dedicated to the management of COVID-19 patients in Romania and could be a better image of AMR changes during the COVID-19 pandemic.

## 4. Materials and Methods

We performed a retrospective study to describe changes in the AMR of *Klebsiella pneumoniae* during the first two years of the COVID-19 pandemic, as compared with 2019; we included all isolates of *Klebsiella pneumoniae* collected from patients with infections or colonization with *Klebsiella pneumoniae* who were admitted to the National Institute of Infectious Diseases “Prof. Dr. Matei Balș”, Bucharest, Romania, between January 2019 and December 2021. All isolates of *Klebsiella pneumoniae* were identified using matrix-assisted laser desorption ionization–time-of-flight mass spectrometry (MALDI-TOF MS) and were analyzed using Biotyper^®^ software version 3.1 (Bruker Daltonik GmbH, Bremen, Germany). Between March 2020 and December 2021, most of the patients were admitted for COVID-19; during this period of time, many bacterial infections were superinfections of COVID-19. The samples were collected from 72 COVID-19 patients in 2020 and 133 COVID-19 patients in 2021. In 2019, we did not have any COVID-19 patients.

In order to eliminate duplicates, we selected only the first isolated sample of *Klebsiella pneumoniae* for each patient. The isolates of *Klebsiella pneumoniae* included in the study were collected from a variety of samples, including blood, urine, pus, sputum, tracheal or bronchial aspirates, and the rectal carriage.

In our analysis, the term CRKP represents isolates of *Klebsiella pneumoniae* with resistance to at least one of tested carbapenems (ertapenem, imipenem, meropenem). In term of resistance to aminoglycosides, we collected data regarding resistance to gentamycin and amikacin. According to EARS-Net, we considered a strain being resistant to aminoglycosides or to fluoroquinolones if the strain was resistant to at least one aminoglycoside, or one fluoroquinolone, respectively. *Klebsiella pneumoniae* isolated are considered to be MDR if they are resistant to at least three classes of antibiotics as well as being susceptible to at least other three classes of antibiotics.

Antibiotic susceptibility was tested using automatic systems (VITEK-2 Compact—bioMérieux S.A., Durham, North Carolina, USA, and MICRONAUT—Bruker Daltonics GmbH & Co., KG, Bremen, Germany). We used antimicrobial resistance breakpoints according to the EUCAST recommendations, versions 9.0 to 11.0, which were applicable in 2019–2021. For all tested antibiotics, where the results of the antibiotic susceptibility testing were given as I (increased exposure), we marked the result as S (susceptible), as defined by 2019 EUCAST recommendations.

Considering that the majority of patients hospitalized in 2020 and 2021 were admitted for COVID-19, we analyzed AMR differences between 2019 and 2020–2021.

We defined the term recent antibiotic consumption/usage as use of antibiotics in the last 6 months before admission in our hospital, and we defined the term recent contact with hospital settings as hospitalization or admission to a nursing home in the last 6 months before admission in our hospital.

The data were collected from the hospital’s electronic database and were analyzed using SPSS Statistics 26 software and MedCalc 22 statistical software. We used a Kolmogorov–Smirnov test for distributions, a one-way ANOVA for continuous variables, a Chi square test and Fisher’s exact test for categorical variables, relative risk for appreciating risk factors, and Z scores for proportions. We considered the results to be statistically significant at a *p* value < 0.05, as measured by two-tailed tests.

## 5. Conclusions

Older patients, recent antibiotic usage, recent contact with hospital settings, and extended hospital stays are risk factors for MDR *Klebsiella pneumoniae*. The rates of resistance for *Klebsiella pneumoniae* are increasing for third-generation cephalosporins, carbapenems, aminoglycosides, and fluoroquinolones, while the rates of resistance to TMP/SMX are decreasing. Therefore, TMP/SMX should be considered as a treatment option in infections produced by carbapenem-resistant or MDR *Klebsiella pneumoniae*. As antimicrobial consumption was found to be a risk factor for MDR *Klebsiella pneumoniae*, we consider that decreasing the excessive use of antibiotics, along with the implementation of measures that restrict pathogen transmission in healthcare settings, is mandatory for preventing further increases in AMR rates. During COVID-19 pandemic, our hospital registered an increase in the number of bacterial HAIs, which consecutively impacted the AMR profiles of *Klebsiella pneumoniae*. Considering that the patients admitted since 2022 were hospitalized not only for COVID-19 but also for bacterial infections, we will consider tracking the further evolution of the AMR rates of *Klebsiella pneumoniae* in the post-pandemic years.

## Figures and Tables

**Table 1:** AMR profiles’ distribution for the isolates of *Klebsiella pneumoniae* included in the study.

AMR Profile	Inpatients (*N*, %) ^2^	Outpatients (*N*, %) ^3^	Z Score
Non-MDR *Klebsiella pneumoniae*	191 (35%)	100 (62.1%)	z = 6.74, *p* < 0.01
CRKP	207 (40.1%)	25 (16.1%)	z = 4.78, *p* < 0.01
MDR *Klebsiella pneumoniae*	307 (56.3%)	59 (36.6%)	z = 3.89, *p* < 0.01
Isolates for which the AMR profile could not be defined ^1^	47	2	-

AMR = antimicrobial resistance. MDR = multidrug-resistant. CRKP = carbapenem-resistant *Klebsiella pneumoniae*. ^1^ There are 48 isolates of *Klebsiella pneumoniae* (mainly from rectal carriage) for which the antimicrobial susceptibility were not tested (for which we only have information about carbapenem resistance), and there was one isolate with only four tested antibiotics within antibiotic susceptibility testing; therefore, the AMR profile could not be defined. ^2^ Number and percentage of each AMR profile of *Klebsiella pneumoniae* from the total number of isolates in inpatients. ^3^ Number and percentage of each AMR profile of *Klebsiella pneumoniae* from the total number of isolates in outpatients.

**Table 2:** AMR profiles’ associations with sample collection day for isolates of *Klebsiella pneumoniae* collected from inpatients.

AMR Profile	≤3 Days (*N* = 306; %) ^1^	3–10 Days (*N* = 99; %) ^2^	≥10 Days (*N* = 140; %) ^3^	Chi Square Test
CRKP	60 (19.6%)	54 (54.5%)	93 (66.4%)	ꭓ^2^ = 104.27, *p* < 0.01
MDR *Klebsiella pneumoniae*	137 (44.7%)	65 (65.6%)	105 (75%)	ꭓ^2^ = 49.68, *p* < 0.01

AMR = antimicrobial resistance. MDR = multidrug-resistant. CRKP = carbapenem-resistant *Klebsiella pneumoniae.* ^1^ Number and percentage of CRKP or MDR isolates of *Klebsiella pneumoniae* collected during the first 3 days of hospitalization. ^2^ Number and percentage of CRKP or MDR isolates of *Klebsiella pneumoniae* collected between 3 and 10 days of hospitalization. ^3^ Number and percentage of CRKP or MDR isolates of *Klebsiella pneumoniae* collected after more than 10 days of hospitalization.

**Table 3:** Associations between age groups and AMR profiles of *Klebsiella pneumoniae.*

Age Group	CRKP		MDR *Klebsiella pneumoniae*	
	R/T, % ^1^	Reference—0–20 Years Age Group (RR, 95% CI)	R/T, % ^2^	Reference—0–20 Years Age Group (RR, 95% CI)
≤20 years	6/35 (17.1%)	RR = 1	13/33 (39.3%)	RR = 1
21–40 years	24/92 (26%)	RR = 1.52, 95% CI [0.68, 3.4]	35/93 (37.6%)	RR = 0.95, 95% CI [0.58, 1.57]
41–60 years	69/174 (39.6%)	RR = 2.31, 95% CI [1.09, 4.9]	92/168 (54.7%)	RR = 1.39, 95% CI [0.89, 2.16]
61–80 years	110/304 (36.1%)	RR = 2.11, 95% CI [1, 4.43]	182/298 (61%)	RR = 1.55, 95% CI [1, 2.38]
≥81 years	23/65 (35.3%)	RR = 2.06, 95% CI [0.92, 4.58]	44/65 (67.6%)	RR = 1.71, 95% CI [1.08, 2.7]

AMR = antimicrobial resistance. MDR = multidrug-resistant. CRKP = carbapenem-resistant *Klebsiella pneumoniae*. R = resistant. T = total. ^1^ Number (as resistant/total) and percentage of CRKP isolates in each age group. ^2^ Number (as resistant/total) and percentage of MDR *Klebsiella pneumoniae* isolates in each age group.

**Table 4:** Recent antibiotic usage and recent contact with hospital settings’ associations with AMR profiles of *Klebsiella pneumoniae.*

Risk Factor	CRKP		MDR *Klebsiella pneumoniae*	
	*N* = 207, % ^1^	Reference— Non-CRKP (RR, 95% CI)	*N* = 307, % ^1^	Reference— Non-MDR (RR, 95% CI)
Recent antibiotic usage	70 (33.8%)	RR = 1.27, 95% CI [1.03, 1.58]	110 (35.8%)	RR = 1.38, 95% CI [1.21, 1.57]
Recent contact with hospital settings	117 (56.5%)	RR = 1.13, 95% CI [0.91, 1.4]	197 (64.1%)	RR = 1.54, 95% CI [1.32, 1.8]

AMR = antimicrobial resistance. MDR = multidrug-resistant. CRKP = carbapenem-resistant *Klebsiella pneumoniae.* ^1^ Number and percentage of isolates collected from patients with recent antibiotic usage or recent contact with hospital settings in each AMR profile of *Klebsiella pneumoniae*.

**Table 5:** Evolution of AMR of *Klebsiella pneumoniae* between 2019 and 2021.

Antibiotic	Resistance (R/T, %)	2019 vs. 2020	2020 vs. 2021	2019 vs. 2021	2019 vs. 2020–2021
C3G	2019—183/364 (50.3%) 2020—81/137 (59.1%) 2021—95/154 (61.7%)	ꭓ^2^ = 3.12, *p* = 0.08	ꭓ^2^ = 0.19, *p* = 0.71	ꭓ^2^ = 5.66, *p* = 0.02	50.3% vs. 60.5% ꭓ^2^ = 6.8, *p* < 0.01
Carbapenems	2019—80/366 (21.9%) 2020—47/138 (34.1%) 2021—105/166 (63.3%)	ꭓ^2^ = 7.91, *p* < 0.01	ꭓ^2^ = 25.69, *p* < 0.01	ꭓ^2^ = 86.27, *p* < 0.01	21.9% vs. 50% ꭓ^2^ = 58.1, *p* < 0.01
MBL	2019—3/79 (3.8%) 2020—17/45 (37.8%) 2021—37/89 (41.6%)	ꭓ^2^ = 24.47, *p* < 0.01	ꭓ^2^ = 0.17, *p* = 0.71	ꭓ^2^ = 32.92, *p* < 0.01	3.8% vs. 40.3% ꭓ^2^ = 33.78, *p* < 0.01
AG	2019—106/366 (29%) 2020—63/137 (46%) 2021—84/153 (54.9%)	ꭓ^2^ = 12.94, *p* < 0.01	ꭓ^2^ = 2.29, *p* = 0.15	ꭓ^2^ = 31.28, *p* < 0.01	29% vs. 50.7% ꭓ^2^ = 32.24, *p* < 0.01
FQ	2019—182/366 (49.7%) 2020—75/137 (54.7%) 2021—96/148 (64.9%)	ꭓ^2^ = 1, *p* = 0.36	ꭓ^2^ = 3.03, *p* = 0.09	ꭓ^2^ = 9.72, *p* < 0.01	49.7% vs. 60% ꭓ^2^ = 6.81, *p* = 0.01
TMP/SMX	2019—165/361 (45.7%) 2020—60/137 (43.8%) 2021—43/152 (28.3%)	ꭓ^2^ = 0.14, *p* = 0.76	ꭓ^2^ = 7.55, *p* < 0.01	ꭓ^2^ = 13.46, *p* < 0.01	45.7% vs. 35.6% ꭓ^2^ = 6.71, *p* = 0.01
Colistin	2019—57/327 (17.4%) 2020—21/90 (23.3%) 2021—60/95 (63.2%)	ꭓ^2^ = 1.61, *p* = 0.22	ꭓ^2^ = 29.78, *p* < 0.01	ꭓ^2^ = 76.81, *p* < 0.01	17.4% vs. 43.8% ꭓ^2^ = 41.67, *p* < 0.01
Tigecycline ^1^	2019—1/328 (0.3%) 2020—2/42 (4.8%) 2021—5/21 (23.8%)	ꭓ^2^ = 9.19, *p* = 0.03	ꭓ^2^ = 5.14, *p* = 0.03	ꭓ^2^ = 64.53, *p* < 0.01	0.3% vs. 11.1% ꭓ^2^ = 30.79, *p* < 0.01
Fosfomycin	2019—99/301 (32.9%) 2020—33/107 (30.8%) 2021—22/26 (84.6%)	ꭓ^2^ = 0.15, *p* = 0.72	ꭓ^2^ = 24.94, *p* < 0.01	ꭓ^2^ = 27.46, *p* < 0.01	32.9% vs. 41.4% ꭓ^2^ = 2.88, *p* = 0.1
MDR	2019—188/366 (51.4%) 2020—82/137 (59.9%) 2021—96/154 (62.3%)	ꭓ^2^ = 2.88, *p* = 0.1	ꭓ^2^ = 0.18, *p* = 0.71	ꭓ^2^ = 5.26, *p* = 0.02	51.4% vs. 61.2% ꭓ^2^ = 6.31, *p* = 0.01

AMR = antimicrobial resistance. MBL = metallo-beta-lactamases. G3C = third-generation cephalosporins. AG = aminoglycosides. FQ = fluoroquinolones, TMP/SMX = trimethoprim/sulfamethoxazole. MDR = multidrug-resistant. R = resistant. T = total. ^1^ In the EUCAST recommendations, there is no established MIC breakpoint of resistance to tigecycline for *Klebsiella pneumoniae*. In these circumstances, we used the PK-PD breakpoint of MIC > 0.5 mg/L for tigecycline. MIC = minimum inhibitory concentration. PK = pharmacokinetics. PD = pharmacodynamics.

**Table 6:** Evolution of AMR of CRKP between 2019 and 2021.

Antibiotic	Resistance (R/T, %)	2019 vs. 2020	2020 vs. 2021	2019 vs. 2021	2019 vs. 2020–2021
TMP/SMX	2019—57/77 (74%) 2020—31/45 (68.9%) 2021—34/87 (39.1%)	ꭓ^2^ = 0.37, *p* = 0.53	ꭓ^2^ = 10.54, *p* < 0.01	ꭓ^2^ = 20.19, *p* < 0.01	57/77 vs. 65/132 74% vs. 49.2% ꭓ^2^ = 12.29, *p* < 0.01
Colistin	2019—41/75 (54.7%) 2020—20/40 (50%) 2021—60/75 (80%)	ꭓ^2^ = 0.22, *p* = 0.69	ꭓ^2^ = 11.08, *p* < 0.01	ꭓ^2^ = 10.94, *p* < 0.01	41/75 vs. 80/115 54.7% vs. 69.6% ꭓ^2^ = 4.35, *p* = 0.04
Tigecycline ^1^	2019—0/73 (0%) 2020—2/13 (15.4%) 2021—5/19 (26.3%)	ꭓ^2^ = 11.49, *p* = 0.02	ꭓ^2^ = 0.54, *p* = 0.67	ꭓ^2^ = 20.31, *p* < 0.01	0/73 vs. 7/32 0% vs. 21.9% ꭓ^2^ = 17.1, *p* < 0.01
Fosfomycin	2019—33/60 (55%) 2020—15/33 (45.5%) 2021—18/20 (90%)	ꭓ^2^ = 0.77, *p* = 0.39	ꭓ^2^ = 10.51, *p* < 0.01	ꭓ^2^ = 7.95, *p* < 0.01	33/60 vs. 33/53 55% vs. 62.3% ꭓ^2^ = 0.61, *p* = 0.45

AMR = antimicrobial resistance. CRKP = carbapenem-resistant *Klebsiella pneumoniae*. R = resistant. T = total. TMP/SMX = trimethoprim/sulfamethoxazole. ^1^ In the EUCAST recommendations, there is no established MIC breakpoint of resistance to tigecycline for *Klebsiella pneumoniae*. In these circumstances, we used the PK-PD breakpoint of MIC > 0.5 mg/L for tigecycline. MIC = minimum inhibitory concentration. PK = pharmacokinetics. PD = pharmacodynamics.

## Data Availability

The data presented in this study are available on request from the corresponding author.
